# Changes in left atrial function following two regimens of combined exercise training in patients with ischemic cardiomyopathy: a pilot study

**DOI:** 10.3389/fcvm.2024.1377958

**Published:** 2024-05-07

**Authors:** Giuseppe Caminiti, Maurizio Volterrani, Ferdinando Iellamo, Giuseppe Marazzi, Vincenzo Manzi, Valentino D’Antoni, Sara Vadalà, Deborah Di Biasio, Matteo Catena, Valentina Morsella, Marco Alfonso Perrone

**Affiliations:** ^1^ Department of Human Science and Promotion of Quality of Life, San Raffaele Open University, Rome, Italy; ^2^ Cardiology Rehabilitation Unit, IRCCS San Raffaele, Rome, Italy; ^3^ Division of Cardiology and Sports Medicine, Department of Clinical Sciences and Translational Medicine, University of Rome Tor Vergata, Rome, Italy; ^4^ Department of Wellbeing, Nutrition and Sport, Pegaso Open University, Naples, Italy

**Keywords:** combined training, atrial dysfunction, ischemic cardiomyopathy, atrial remodeling, cardiac rehabilitation

## Abstract

**Purpose:**

Left atrial dysfunction has shown to play a prognostic role in patients with ischemic cardiomyopathy (ICM) and is becoming a therapeutic target for pharmacological and non-pharmacological interventions. The effects of exercise training on the atrial function in patients with ICM have been poorly investigated. In the present study, we assessed the effects of a 12-week combined training (CT) program on the left atrial function in patients with ICM.

**Methods:**

We enlisted a total of 45 clinically stable patients and randomly assigned them to one of the following three groups: 15 to a supervised CT with low-frequency sessions (twice per week) (CTLF); 15 to a supervised CT with high-frequency sessions (thrice per week) (CTHF); and 15 to a control group following contemporary preventive exercise guidelines at home. At baseline and 12 weeks, all patients underwent a symptom-limited exercise test and echocardiography. The training included aerobic continuous exercise and resistance exercise. The analysis of variance (ANOVA) was used to compare within- and inter-group changes.

**Results:**

At 12 weeks, the CTLF and CTHF groups showed a similar increase in the duration of the ergometric test compared with the control (ANOVA *p* < 0.001). The peak atrial longitudinal strain significantly increased in the CTHF group, while it was unchanged in the CTLF and control groups (ANOVA *p* = 0.003). The peak atrial contraction strain presented a significant improvement in the CTHF group compared with the CTLF and control groups. The left ventricular global longitudinal strain significantly increased in both the CTHF and the CTLF groups compared with the control group (ANOVA *p *= 0.017). The systolic blood pressure decreased in the CTHF and CTLF groups, while it was unchanged in the control group. There were no side effects causing the discontinuation of the training.

**Conclusions:**

We demonstrated that a CT program effectively improved atrial function in patients with ICM in a dose–effect manner. This result can help with programming exercise training in this population.

## Introduction

Ischemic cardiomyopathy (ICM) is characterized by a remodeling process that is triggered by an ischemic insult to the left ventricle (LV) that impairs LV relaxation, raises LV stiffness, and ultimately causes the increase of LV filling pressure (1). In many patients with ICM, the remodeling also extends to the left atrium (LA) because this chamber takes part in the LV filling process during the diastolic phase (2, 3). The LA remodeling process is an electro-mechanical process that culminates in LA enlargement and can be associated with the onset of atrial fibrillation. According to the current model, structural changes in the LA are preceded by functional abnormalities of this chamber (4). The onset of LA functional abnormalities is considered the first sign of an increased LV filling pressure because it can be detected even before the other indices of diastolic dysfunction appear (5). A two-dimensional speckle-tracking analysis performed during an echocardiography examination can assess LA function by identifying the reservoir, conduit, and contraction phases (6). Several studies demonstrated that the reservoir strain, which is often expressed as the peak atrial longitudinal strain (PALS), is impaired in a wide spectrum of cardiovascular conditions, with the highest degree of dysfunction being observed in patients with heart failure (HF) with both reduced and preserved ejection fractions (EF) (7). The impairment of PALS has been associated with a reduced exercise tolerance (8), a greater risk of atrial fibrillation insurgency (9), and a poor outcome (10, 11). Similarly to PALS, the peak atrial contraction strain (PACS), reflecting the booster phase of the LA, has also recently been shown to predict survival in patients with cardiovascular diseases (12). In light of the prognostic role of its component, LA dysfunction has recently become a therapeutic target. In this regard, some preliminary studies have provided evidences on the possibility of counteracting the LA remodeling process through the improvement of LA functional parameters in different clinical contexts. This goal has been achieved independently through either the administration of sacubitril–valsartan in patients with advanced HF with reduced ejection fraction (HFrEF) (13) or weight loss in diabetic obese subjects (14). Exercise training, mainly in the setting of supervised cardiac rehabilitation programs, exerts several favorable effects in patients with ICM, such as it reduces symptoms, enhances exercise tolerance, and ultimately improves the prognosis of these patients (15, 16). Therefore, practicing regular exercise training is strongly recommended by the current guidelines for ICM (17). Among different exercise modalities, combined training (CT) is particularly attractive for patients with ICM compared with aerobic continuous exercise alone because it carries additional advantages on several parameters, including metabolism, muscle strength and bulk, and blood pressure (BP) variability (18–20). In patients with ICM, exercise training causes “central” cardiac effects that can help stabilize clinical conditions and improve prognosis (21). Although exercise training is an accepted strategy for attenuating LV remodeling in ICM (22, 23), less attention has been given to the LA. Improvement of the LA reservoir and contraction strains has recently been observed in patients with HF and mildly reduced EF (HFmrEF) after 12 weeks of CT (24). However, it remains to be established whether or not CT has beneficial effects on the LA function in patients with asymptomatic ICM which has not yet developed HF. The purpose of this study was to compare the changes in the left atrial function produced by two regimens of CT vs. control in patients with ICM. The primary endpoint was the comparative changes in PALS in patients undergoing supervised CT programs vs. control. The secondary endpoint was to compare the changes in PALS produced by the two different regimens of CT.

## Methods

### Population

The study enrolled 45 patients of both genders with a previous diagnosis of ICM who had never experienced signs and/or symptoms of heart failure. The patients were recruited if they were older than 45 years old. The diagnosis of ICM was established if the subjects had one or more of the following clinical conditions: previous ST-elevation myocardial infarction (STEMI); no ST-elevation myocardial infarction (NSTEMI)/unstable angina; percutaneous coronary intervention (PCI); and coronary artery bypass grafting (CABG). The subjects were enrolled if they were in stable clinical condition, were not hospitalized in the previous 6 months, and their pharmacological therapy was unchanged in the previous 3 months. We implemented the following exclusion criteria: myocardial ischemia or threatening arrhythmias during the resting assessment or ergometric test; previous HF diagnosis; permanent atrial fibrillation; baseline blood pressure levels at rest exceeding 160/100 mmHg; severe heart valve diseases; hypertrophic cardiomyopathy diagnosis; anemia with hemoglobin levels below 10.5 g/dl; concomitant diagnosis of chronic respiratory disease with a documented FEV1 below 50%; and previous peripheral artery disease diagnosis with exercise-limiting claudication. Moreover, we excluded patients who had participated in training programs in the previous 6 months or who reported engaging in regular exercise spontaneously. The study complied with the Declaration of Helsinki and was approved by the local ethics committee of San Raffaele IRCCS (protocol number 18/2022). All patients provided written informed consent before participating in the study.

### Study design

**Figure 1:**
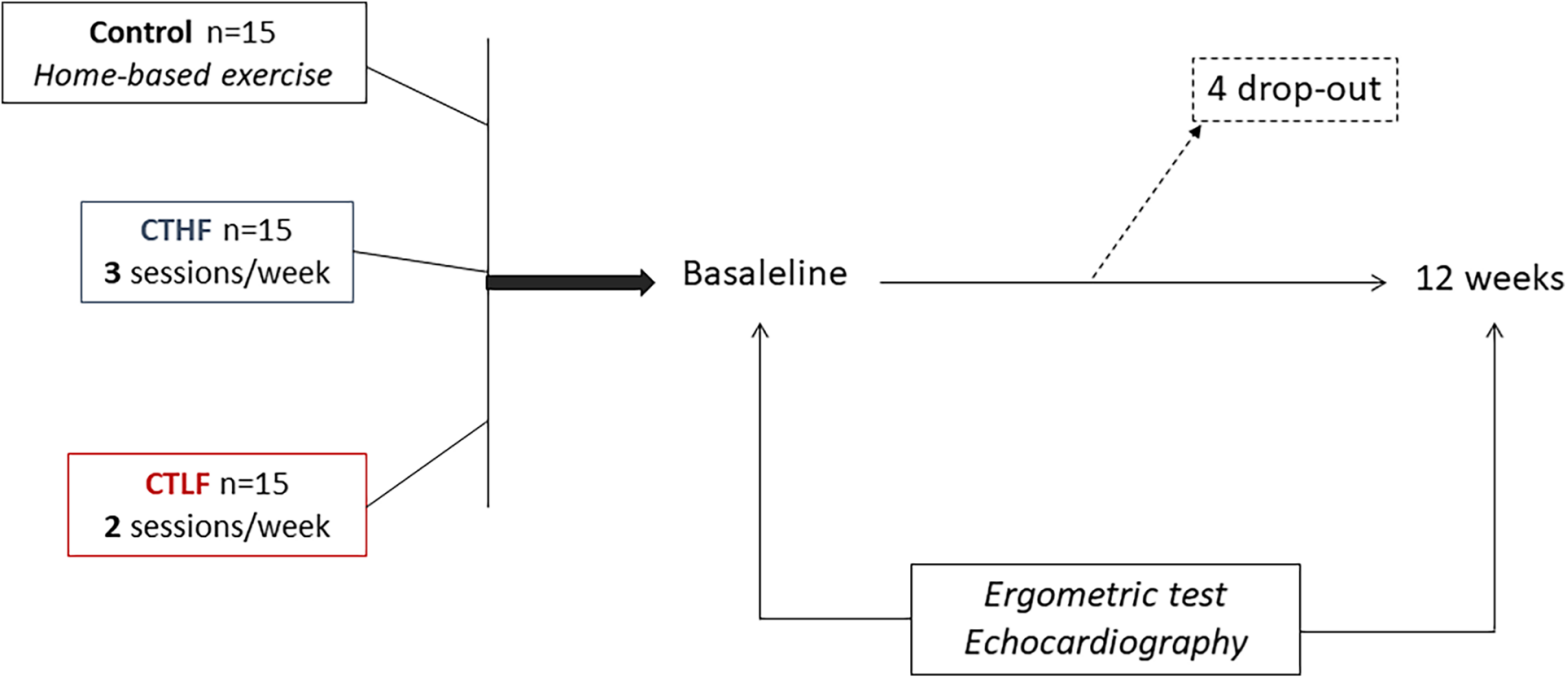
Study flowchart.

The study design has been summarized in Figure 1. It was conceived as a pilot study with three parallel arms. The patients were randomly assigned on a 1:1:1 basis to one of the following groups: (1) the combined training high-frequency (CTHF) group; (2) the combined training low-frequency (CTLF) group; and (3) the control group. Each group was composed of 15 patients. The randomization code was developed by a computer random-number generator to select random permuted blocks. The patients belonging to the CTHF and CTLF groups performed supervised exercise training sessions in the rehabilitation facility of San Raffaele IRCCS in Rome, while patients belonging to the control group were discharged and given instructions to perform exercises at home according to the guideline recommendations (16). At baseline, the patients underwent a preliminary visit, during which their medical histories and anthropometric parameters were collected. The body mass index (BMI) was calculated using the following formula: BMI = kg/m^2^, where kg is the weight of the patient in kilograms and m^2^ is their height in meters squared. The patients who met the inclusion/exclusion criteria and provided consent for the study were summoned in another day (i.e., within a week from the first visit). During the second visit, they underwent echocardiography and an ergometric test. The examinations were repeated at 12 weeks.

### Transthoracic echocardiography

The echocardiography was performed with patients in the supine position. An experienced sonographer, blinded to the type of patient's allocation, performed echocardiography at baseline and 12 weeks on all study subjects. A cardiovascular ultrasound Vivid E95® (GE HealthCare, Chicago, IL, USA) with a 4.0-MHz transducer was used. During echocardiography, one-lead electrocardiography monitoring placed on the chest was conducted. After the acquisition, all echocardiographic images were digitally stored and analyzed offline. During the review process, an experienced technician, who was also blinded to all participants’ details, performed deformation measures using a proprietary software (version 10.8, EchoPAC; GE Vingmed Ultrasound, Norway). The left ventricular end-diastolic volume (LVEDV) and left ventricular end-systolic volume (LVESV) were measured from the apical two- and four-chamber windows. The LVEF was then calculated using the modified Simpson method. The LA volume was measured from standard apical four-chamber and three-chamber views at end systole, before the opening of the mitral valve, using the biplane Simpson method of disks. The LA volume index (LAVI) was calculated by dividing the LA volume by the body surface area of the patients. The E/A ratio was defined as the ratio of the peak left ventricle filling velocity (E wave) in early diastole, corresponding to atrial relaxation, to the peak velocity flow in late diastole (A wave), corresponding to atrial contraction. The LV E/E′ ratio was calculated as the ratio between the E wave velocity and the average between the septal and lateral LV E′ wave velocities. Color tissue Doppler tracings were performed in the four-chamber view, with the range gate placed at the lateral mitral annular segments. The LV global longitudinal strain (GLS) was measured through the two-, three-, and four-chamber views. The LV endocardial boundary was automatically detected by the software; however, when deemed appropriate, it was edited to conform to the visualized LV boundaries. The maximum negative value of strain during systole measured by the software represented the maximum contractility for each segment. The average of the values from each segment was then calculated to determine the LVGLS. The LA strain was measured through the two- and four-chamber views. LA deformation tracking was carried out using the R wave as the starting point (R–R gating). The endocardial and epicardial contours of the LA were traced using an automatic contour tracking algorithm. Manual adjustments were also made when necessary. The automatic algorithm placed a set of control points on the middle curve of the myocardial wall in the reference phase based on the drawn endocardial and epicardial contours. The longitudinal strain curves were generated for each segment by the software program. The mean curve of all segments was then calculated. The LA reservoir strain, conduit strain, and contractile strain were obtained from the subdivision of longitudinal strain measurements (25). PALS was measured as a positive peak during LV systole at the end of the atrial diastolic phase, and PACS was measured as a positive peak during early LV diastole, right before the start of the atrial systolic phase (26).

### Ergometric test

The ergometric test was performed on a cycle ergometer (Mortara Instrument, Casalecchio Di Reno, Italy). The heart rate and BP were measured at rest, before starting the exercise, at each stage of the exercise, and every 2 min during the recovery phase. The test was terminated if the patient experienced muscle exhaustion and chest pain or expressed the desire to stop the test. At each stage of the exercise protocol, the patients were asked to rate their perceived sensation of fatigue according to the modified Borg scale (27).

### Exercise training protocol

The exercise training sessions were performed in the cardiac rehabilitation facility of San Raffaele IRCCS in Rome. The patients were asked to continue their usual dietary and lifestyle habits for the entire duration of the exercise training program. All exercise sessions were held in the morning, between 8 and 11 AM, and were supervised by two physiotherapists who advised patients regarding the setup of treadmills, cycles, and dynamometers through the training sessions and ensured that they performed the exercises correctly. The exercise sessions were also supervised by a physician specialized in sport medicine and with experience in the cardiac rehabilitation field, and by a nurse. During the first exercise session, we monitored the patients’ heart rhythms for safety using telemetry. The duration of each exercise session was 60 min. Before starting the sessions, the patients performed a 10-min warm-up, and at the end of each session, they performed a 10-min cool-down. During each session, the patients first performed the aerobic exercises before the resistance exercises. For both the CTHF and CTLF groups, the exercise sessions were planned as follows: 40 min of aerobic training on a treadmill or a bike followed by 20 min of resistance training (Technogym Wellness System, Technogym, Cesena, Italy). The rate of perceived exertion (RPE) method was used to plan the intensity of the aerobic component over the whole training program. The patients were asked to reach an intensity target of 13–14 (somewhat hard). To retain the patients’ level of effort during the entire training period, they were left free to change the treadmill/cycle setup in subsequent sessions. The RPE method was chosen to permit updating the exercise prescriptions as the fitness levels changed. The resistance component of the training sessions consisted of the following exercises: leg press, leg extension, shoulder press, chest press, low row, and vertical traction. The muscle groups involved were the quadriceps, back muscles, deltoids, and biceps. The intensity of each resistance exercise was established through the assessment of the corresponding 1-repetition maximum (1-RM). For each exercise, the patients were required to perform a warm-up set comprising 8–10 repetitions at 60% of their 1-RM, and this intensity was fixed during the entire training program. Then, patients performed one repetition at their maximal effort. This latter was carried out three times, and between each effort, the patients could rest for 2–3 min. The highest value of strength obtained was used as 1-RM (28). The patients performed the 1-RM test at baseline and every 3 weeks to update the exercise loads as the fitness levels changed. The patients performed two sets of every exercise and had 2 min of rest between sets. Each set included eight repetitions. Particular attention was paid to avoiding the contraction of muscle groups other than those specifically involved in the exercise (that is, accessory muscle recruitment). The following formula was adopted to assess the patients’ adherence to the training program: (attended sessions/planned sessions × 100). The control group was advised to perform physical activities at home according to the contemporary guidelines (17). The patients belonging to this group did not receive any supervision or wearable device for performing exercises at home. They only received a training manual and educational materials. The training manual summarized the guideline recommendations about the exercise modalities and the training intensity and frequency. After the enrollment visit at baseline, there were no further contacts between the study investigators and the patients of the control group throughout the study. The participants were finally contacted at 12 weeks and summoned for the final evaluation at our center. During that last visit, they were asked about the number of exercise sessions performed at home.

### Statistical analysis

This research was conceived as a pilot study; thus, a formal sample size calculation was not required. The Shapiro–Wilks hypothesis test was used to check the assumption of normality. The pre-exercise and post-exercise data variables were assessed using a repeated-measure one-way analysis of variance (ANOVA) with Bonferroni corrections for *post-hoc* testing. The Pearson correlation coefficient test was used to measure the strength of the linear association between two variables. The level of significance was set at *p* < 0.05. The data were analyzed using SPSS software (version 20.0 IBM Corp., Armonk, NY, USA). The intra-observer and inter-observer variabilities for the LA strain measures were evaluated in a group of 10 patients. Measurements of the primary and secondary endpoints of this study were repeated by the same observer after an interval of ≥1 week and by a second independent blinded observer. The intraclass correlation coefficient was used to measure reproducibility, with a good agreement designated as >0.80.

## Results

The baseline features of the population are presented in Table 1. At baseline, the three groups were comparable in terms of age, anthropometric and clinical parameters, and pharmacological therapy. Overall, 30 of the 45 patients (66.6%) had a previous STEMI. Of these 30, 21 patients (70.0%) had a previous anterior STEMI. The EF ranged from 60 to 40%. Overall, 11 patients had an EF below 50%. All patients had arterial hypertension and were taking an average of 2.3 ± 1.2 drugs for BP control. Furthermore,17 of the 45 patients (37.7%) were obese (i.e., with a BMI over 30 kg/m^2^), and 19 (42.2%) were overweight (i.e., with a BMI between 25 and 30 kg/m^2^). No side effects occurred during the entire study period. Four patients dropped out of the study, and three of them (i.e., one from the CTHF group, one from the CTLF group, and one from the control group) refused to continue. One patient from the control group needed changes in pharmacological therapy because of uncontrolled high BP values. Altogether 41 patients completed the study and were included in the analysis. The average number of sessions performed by the patients from the CTHF group was 35.1 ± 5.7, whereas that from the CTLF group was 34.6 ± 4.2, with adherence rates of 88.8% and 87.5%, respectively. The patients of the control group declared an average number of sessions of 11.3 ± 4.7. Out of the 15 patients (46.6%), 7 of this group declared that they discontinued their physical activity within the first 2 weeks of the study.

**Table 1:** Baseline features of recruited patients according to the three group allocations.

	CTHF (*n* = 15)	CTLF (*n* = 15)	Control (*n* = 15)
Age, years	63.4 ± 13.2	64.0 ± 11.9	63.7 ± 12.7
BMI, kg/m^2^	27.9 ± 6.4	28.2 ± 7.2	27.5 ± 6.6
Males, *n* (%)	11 (73.3)	10 (66.6)	11/4 (73.3)
Previous STEMI/NSTEMI-UA, *n* (%)	9 (60.0)/7 (46.6)	11/6 (40.0)	10 (66.6)/5 (33.3)
Previous CABG/PCI, *n* (%)	9 (60.0)/12 (80.0)	8 (53.3)/13 (86.6)	7 (46.6)/13 (86.6)
Multivessel disease, *n* (%)	11 (73.3)	11 (73.3)	10 (66.6)
Carotid artery disease, *n* (%)	7 (46.6)	6 (40.0)	6 (40.0)
Hypertension, *n* (%)	13 (86.6)	15 (100.0)	12 (80.0)
Diabetes, *n* (%)	4 (26.6)	3 (20.0)	3 (20.0)
Hypercholesterolemia, *n* (%)	15 (100.0)	14 (93.3)	15 (100.0)
eGFR, ml/min/1.73 m^2^	78.3 ± 11.6	72.8 ± 14.1	75.7 ± 13.8
SBP, mmHg	126.6 ± 34.0	125.8 ± 27.4	126.1 ± 30.9
DBP, mmHg	82.2 ± 14.7	83.6 ± 14.4	82.8 ± 15.3
HR, b/min	67.4 ± 11.6	64.6 ± 13.2	66.1 ± 11.8
NT-proBNP, pg/ml	97.4 ± 11.8	89.9 ± 13.8	94.5 ± 16.2
Echocardiography			
LVEDV, mm^3^/m^2^	66.6.3 ± 22.3	67.9 ± 17.4	68.7 ± 18.8
LVESD, mm^3^/m^2^	36.7 ± 12.3	37.1 ± 14.6	37.6 ± 13.7
LVEF, %	55.3 ± 8.4	54.6 ± 7.2	54.3 ± 11.8
LVGLS, %	−17.3 ± 4.8	−18.5 ± 4.1	−17.7 ± 5.1
E/E′	9.1 ± 2.0	8.7 ± 1.8	8.8–1 ± 2.2
LAVI, mm^3^/m^2^	27.8 ± 7.4	26.6 ± 6.6	28.4 ± 8.7
MR mild/moderate, *n* (%)	8 (53.3)/4 (26.6)	9 (60.0)/5 (33.3)	8 (53.3)/3 (20.0)
PALS, %	14.0 ± 4.6	13.8 ± 2.9	14.2 ± 3.1
PACS, %	12.5 ± 3.9	12.1 ± 3.4	12.6 ± 3.2
Treatment			
ACE-i/ARBs, *n* (%)	14 (93.3)	15 (100.0)	13 (86.6)
Beta-blockers, *n* (%)	14 (93.3)	14 (93.3)	15 (100)
MRAs, *n* (%)	8 (53.3)	6 (40.0)	7 (46.6)
Ivabradine, *n* (%)	4 (26.6)	3 (20.0)	6 (40.0)
Statins, *n* (%)	15 (100.0)	14 (93.3)	13 (86.6)
CCAs, *n* (%)	7 (46.6)	9 (60.0)	7 (46.6)
Acetylsalicylic acid	15 (100.0)	14 (93.3)	14 (93.3)
Clopidogrel	3 (20.0)	4 (26.6)	3 (20.0)

BMI, body mass index; STEMI, ST-segment elevation myocardial infarction; NSTEMI-UA, no ST-segment elevation myocardial infarction—unstable angina; CABG, coronary artery bypass grafting; eGFR, estimated glomerular rate filtration; SBP, systolic blood pressure; DBP, diastolic blood pressure; HR, heart rate; LVEDV, left ventricular end-diastolic volume; LVESV, left ventricular end-systolic volume; LVEF, left ventricular ejection fraction; LVGLS, left ventricular global longitudinal strain; LVGCS, left ventricular global circumferential strain; MR, mitral regurgitation; PALS, peak atrial longitudinal strain; PACS, peak atrial contraction strain; ACE-Is, angiotensin-converting enzyme inhibitors; ARBs, angiotensin receptor blockers; MRAs, mineralocorticoid receptor antagonists; CCAs, calcium channel antagonists.

### Within-group analysis

**Figure 2:**
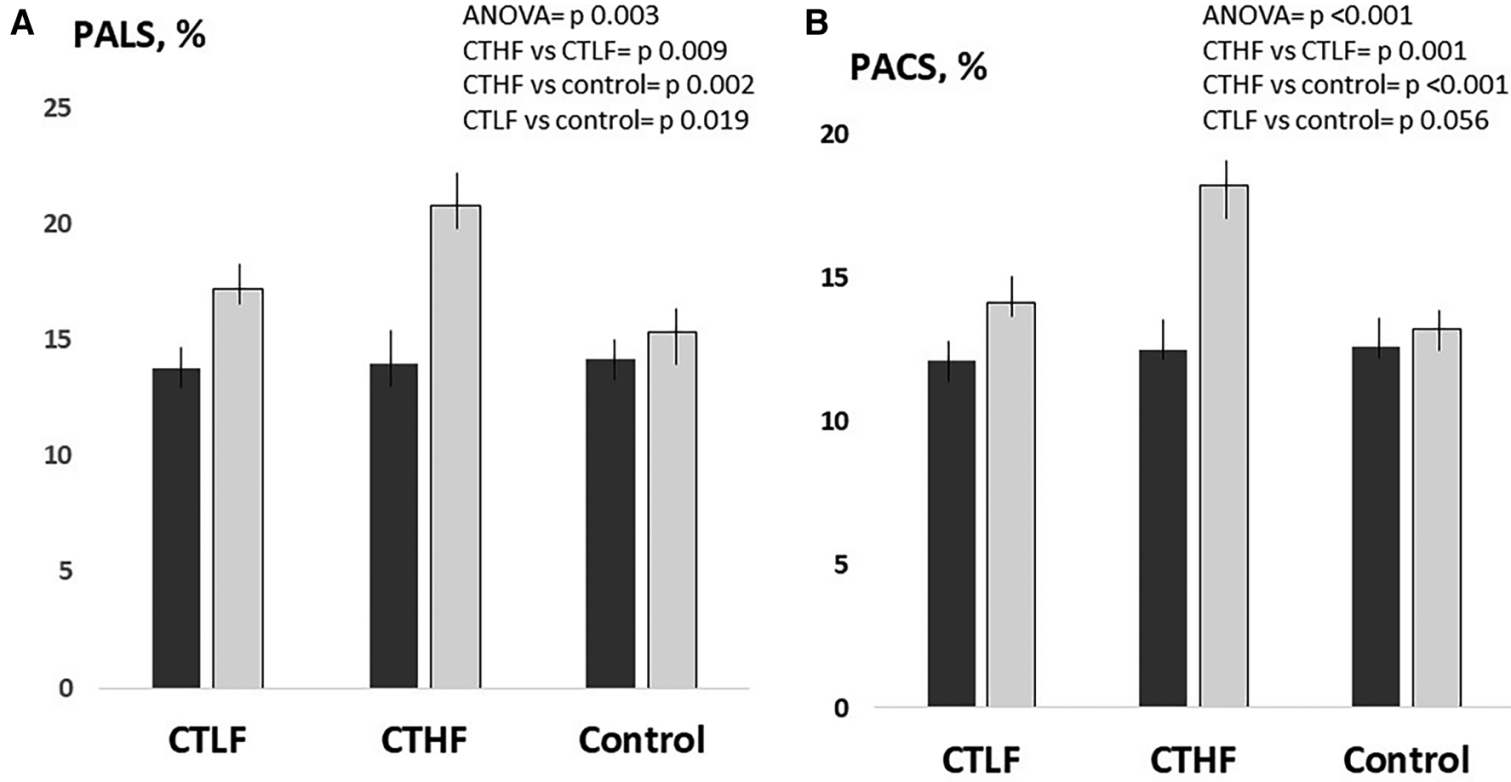
Between-group comparisons at 12 weeks of the changes in PALS (**A**) and PACS (**B**) occurring in the CTLF, CTHF, and control groups. Results of the one-way ANOVA and the Bonferroni *post-hoc* tests. Dark gray bars = baseline; light gray bars = 12 weeks.

**Figure 3:**
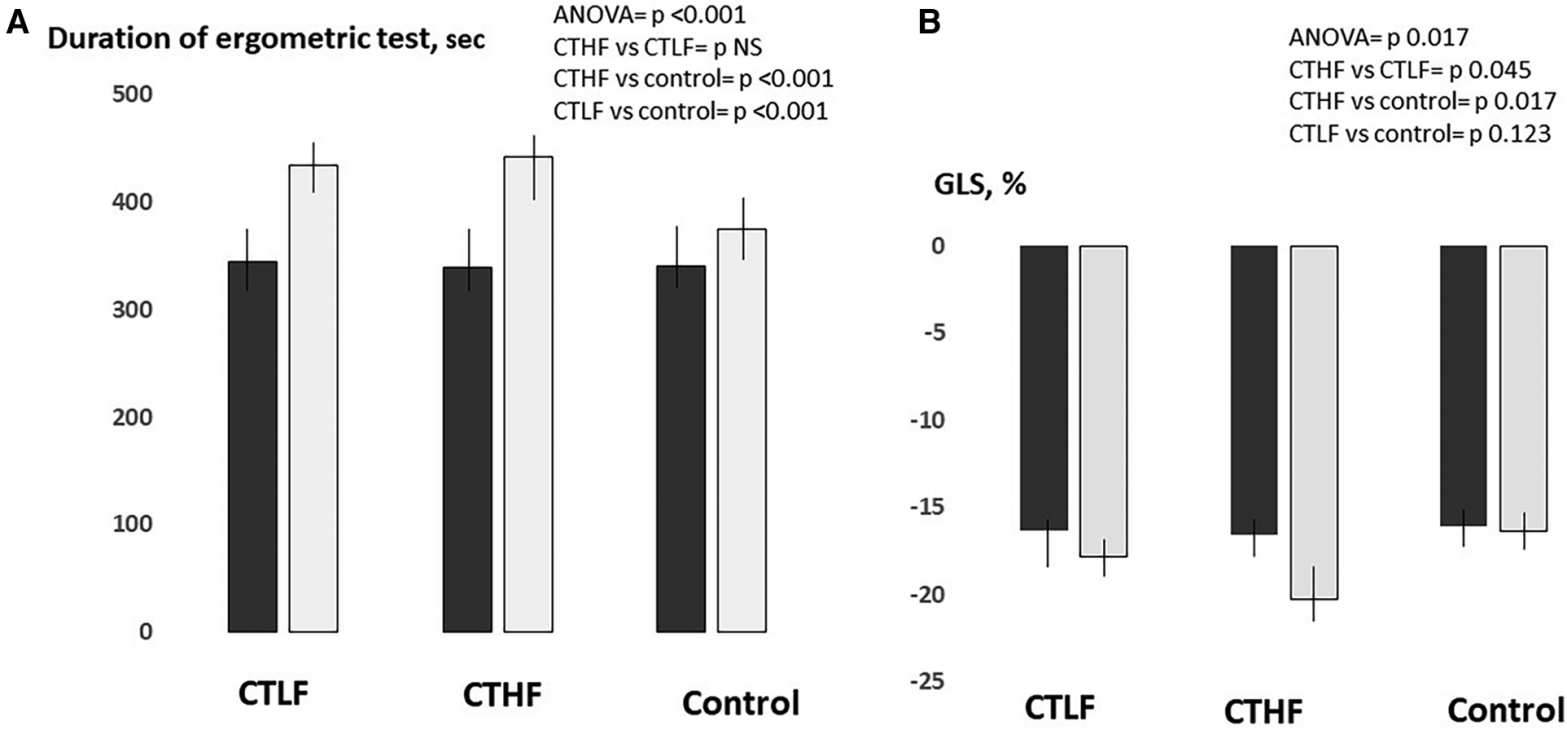
Between-group comparisons of the changes in time during the ergometric test (**A**) and LVGLS (**B**) occurring in the CTLF, CTHF, and control groups at 12 weeks. Results of the one-way ANOVA and Bonferroni *post-hoc* tests. Dark gray bars = baseline; light gray bars = 12 weeks.

**Figure 4:**
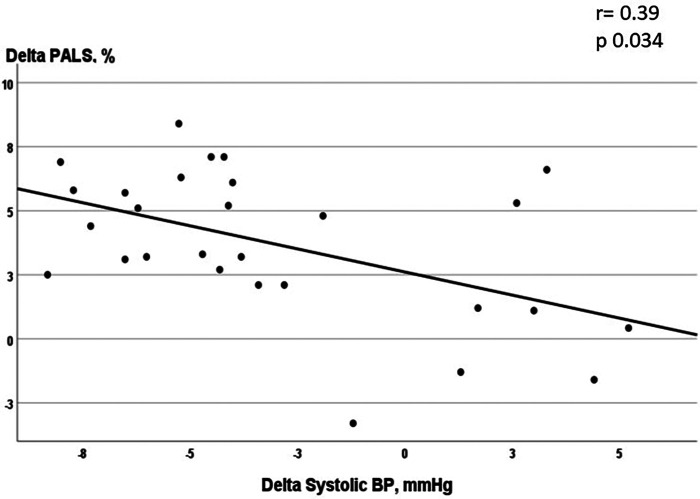
Correlation between the changes in the systolic blood pressure and in PALS in patients undergoing supervised exercise (CTLF + CTHF).

At 12 weeks, the duration of the ergometric test significantly increased in both the CTLF and CTHF groups (+26%, *p* = 0.007, and +30%, *p* = 0.001%, respectively), while it was unchanged in the control group (Figure 2). PALS significantly increased in both the CTLF and CTHF groups (+24.6%; *p* = 0.024, and +48%–5%; *p* = 0.012, respectively), while it was unchanged in the control group (Figure 3). PACS significantly increased in the CTHF group (+44.0%), but remained unchanged in the CTLF and control groups. The E/E′ ratio, LAVI, LVEDV, LVESV, and diastolic BP did not change in any of the three groups (Table 2). The left ventricular global longitudinal strain (LVGLS) significantly improved in the CTHF group (−22.3%; *p* = 0.037) but remained unchanged in the CTLF and control groups (Figure 4). The systolic BP significantly decreased in the CTHF and CTLF groups (−9.1%; *p* = 0.012, and −4.4%; *p* = 0.036, respectively), while it remained unchanged in the control group.

**Table 2:** Changes in the hemodynamic and echocardiography parameters in the three study groups.

	CTLF (*n *= 15)		CTHF (*n* = 15)		Control (*n* = 15)		ANOVA *p*
	Baseline	12 weeks	Baseline	12 weeks	Baseline	12 weeks	
Resting HR, b/min	67.4 ± 11.6	64.3 ± 17.4*	64.6 ± 13.2	60.0 ± 15.3*	66.1 ± 11.8	67.1 ± 20.5	0.034
Resting SBP, mmHg	126.6 ± 34.0	121.1 ± 44.7*	125.8 ± 27.4	115.6 ± 39.0*	126.1 ± 30.9	125.5 ± 42.1	0.028
Resting DBP, mmHg	82.2 ± 14.7	80.2 ± 16.7	83.6 ± 14.4	82.2 ± 20.4	82.8 ± 15.3	81.3 ± 16.4	0.311
Time at ergometric test, s	345.5 ± 71.8	434.6 ± 88.2*	339.0 ± 96.4	442.9 ± 74.8*	341 ± 93.5	375 ± 86.1	<0.001
Echocardiography							
LVEDV, mm^3^/m^2^	66.6 ± 22.3	67.8 ± 17.4	67.9 ± 19.5	68.3 ± 24.0	68.7 ± 18.8	68.3 ± 26.3	0.211
LVESV, mm^3^/m^2^	36.7 ± 12.3	37.0 ± 14.5	37.1 ± 14.6	36.8 ± 12.7	37.6 ± 13.7	36.8 ± 11.1	0.141
LVEF, %	55.3 ± 8.4	54.6 ± 7.9	54.6 ± 7.2	54.1 ± 8.8	54.3 ± 11.8	54.1 ± 8.4	0.152
LVGLS, %	−17.3 ± 4.8	−18.8 ± 5.5	−18.5 ± 4.1	−21.8 ± 6.7*^,^**	−17.7 ± 5.1	−17.9 ± 5.3	0.017
E, cm/s	57.5 ± 14.3	58.3 ± 17.0	58.7 ± 18.9	60.3 ± 11.8	57.8 ± 11.8	59.7 ± 16.3	0.242
A, cm/s	59.0 ± 16.1	60.4 ± 15.1	62.6 ± 16.3	61.8 ± 13.5	61.2 ± 17.4	60.9 ± 15.4	0.286
E/A ratio	0.97 ± 0.3	0.93 ± 0.5	0.94 ± 0.5	0.96 ± 0.4	0.94 ± 0.7	0.93 ± 0.3	0.175
E′, cm/s	6.3 ± 1.3	6.7 ± 2.2	6.7 ± 1.7	7.0 ± 3.1	6.5 ± 2.2	6.9 ± 2.6	0.138
E/E′ ratio	9.1 ± 2.0	8.7 ± 1.8	8.7 ± 1.8	8.6 ± 2.4	8.8 ± 1.8	8.7 ± 3.0	0.187
TRV, m/s	1.8 ± 0.6	1.7 ± 0.6	1.9 ± 1.6	1.7 ± 0.6	1.7 ± 0.9	1.7 ± 1.1	0.295
PALS, %	14.0 ± 4.6	17.2 ± 6.1*	13.8 ± 2.9	19.8 ± 6.4*^,^**	14.2 ± 3.1	15.0 ± 4.6	0.003
PACS, %	12.5 ± 3.9	14.1 ± 5.1	12.1 ± 3.4	18.2 ± 3.9*^,^**	12.6 ± 3.2	13.2 ± 4.8	<0.001
LAVI, ml/m^2^	27.8 ± 7.4	26.4 ± 5.8	26.6 ± 6.6	25.3 ± 5.0	28.4 ± 8.7	27.9 ± 6.2	0.435

HR, heart rate; SBP, systolic blood pressure; DBP, diastolic blood pressure; LVEDV, left ventricular end-diastolic volume; LVESV, left ventricular end-systolic volume; LVEF, left ventricular ejection fraction; GLS, global longitudinal strain; TRV, tricuspid regurgitation velocity; PALS, peak atrial longitudinal strain; PACS, peak atrial contraction strain; LAVI, left atrial volume index.

* *p* < 0.05 vs. control.

** *p* < 0.05 vs. the intervention arm.

### Inter-group analysis

The increase in PALS observed in the CTHF group was significantly higher than that in the CTLF [+3.2% (95% CI = 0.8–5.5), *p* = 0.009] and control [+4.7% (95% CI = 1.8–7.5), *p* = 0.002] groups (Figure 2). The increase in PALS in the CTLF group was higher than that in the control [+2.4% (95% CI = 0.4–4.4), *p* = 0.019] group. The increase in PACS in the CTHF group was significantly higher than that in the CTLF [+3.7% (95% CI = 2.1–5.2), *p* = 0.001] and control [+5.0% (95% CI = 3.5–6.5), *p* < 0.001] groups. There were no significant changes in PACS in the CTLF and control groups [+1.3% (95% CI −0.3–2.7), *p* = 0.056]. The changes in the LVGLS in the CTHF group were higher than those in the CTLF [+4.9% (95% CI = 2.3–9.8), *p* = 0.045] and control [+6.0% (95% CI = 1.1–10.8), *p* = 0.017] groups (Figure 3). There were no significant changes in the LVGLS in the CTLF and control groups [+1.0% (95% CI −0.2–2.3), *p *= 0.126]. The increase in the duration of the ergometric test was significantly higher for the CTHF and CTLF groups compared with that in the control group [+68.1 s (95% CI = 51.6–86.5), *p* < 0.001; and +54.6 s (95% CI = 37.5–71.8), *p* < 0.001, respectively], while there were no significant differences between the two CT groups [+14.4 s (95% CI = 2.5–28.3), *p* = 0.053]. There was an inverse significant correlation between the changes in systolic BP and the changes in PALS in patients undergoing supervised CT (*r* = −0.39; *p *= 0.034) (Figure 4).

## Discussion

The present study investigated the effects of two regimens of combined exercise training on the LA function in patients with ICM. The results led to two main findings: first, we observed that both regimens of CT elicited favorable effects on the LA reservoir strain compared with the control group; and second, the regimen with the highest training frequency led to a greater increase of PALS and PACS than that with the lowest frequency of training sessions. Regarding the first point, the LA adaptations to exercise training have been assessed mainly in healthy subjects and athletes, while very few studies on this topic have previously been conducted in patients with cardiovascular diseases. In sedentary healthy individuals, increases in the LA strain parameters have been observed after short exercise training interventions without any detectable change in the LA size (29). Conversely, no changes or small reductions in reservoir strain were described in athletes (30, 31). LA dysfunction has been observed in patients with a wide range of cardiovascular diseases and is interpreted as the first step of the LA remodeling process (4). This process is related to an increase in the LA pressure, which in turn is due to the increase in the LV stiffness and the LV filling pressure following an ischemic insult, and can evolve toward LA enlargement. Trying to revert the LA remodeling process by improving the LA structure and function is becoming an important goal in ICM management. This is because LA size and function have both emerged as prognostic factors in the general population and in patients with ICM (11, 32, 33). Exercise training has well proven the anti-remodeling effects in the post-ischemic LV (34), which are among the mechanisms through which exercise favorably impacts ICM. The potential adaptation of the LA to exercise training in these patients may also have an important clinical value. Preliminary data suggest the possibility of counteracting LA remodeling through exercise training. In a recent research, a regimen of CT, consisting of three sessions/week and lasting 12 weeks, was effective in increasing the reservoir strain and the contraction strain in patients with HFmrEF and underlying ICM (24). The present study confirms the results of the previous research and extends them to patients who are in a less advanced stage of ICM: indeed, in this study we enrolled asymptomatic subjects with normal LA volume, normal E/E′ ratio, with on average normal EF, who had never experienced symptoms or signs of HF. The changes in the LA functional parameters were not coupled with change in the LA size. The lack of effects on LA size could depend on the shortness of the CT program (i.e., lasting only 12 weeks), and we cannot rule out that different results could be obtained through longer exercise interventions. Previous research on the effects of exercise training on the LA size showed mixed results (35, 36). Moreover, an effective exercise protocol for modifying the LA size remains uncertain and is still a debated point (37). This study did not allow us to explore what mechanisms underlie the beneficial effects produced by CT on LA function. However, we found that the systolic BP values decreased, and the LVGLS improved in the CTLF and CTHF groups at 12 weeks. We can hypothesize that the improvements in the LVGLS were secondary to the exercise-induced systolic BP reductions, as demonstrated in other studies involving anti-hypertensive drugs (38). The improvement of the LVGLS may consequently have had positive repercussions on the LA function because the changes in the LVGLS are at least partly responsible for the changes in the LA reservoir strain (39). Therefore, our results seem to suggest that the increase in PALS reflected the improved hemodynamic conditions of the downstream ventricle. In this regard, we interestingly found a significant correlation between the changes in the systolic BP values and those in PALS. New studies are needed to fully understand the clinical implications of the beneficial effect produced by exercise training on the LA function. In particular, it should be assessed whether these effects constitute an additional mechanism by which exercise training contributes to the stabilization of these patients or if they are a consequence of the effects produced by exercise on the left ventricle. The effects of other exercise modalities on the LA parameters should also be tested in future research. In this study, we found that the values of PALS were lower in comparison with those reported by the literature in healthy age-matched subjects and were instead comparable with those that we and other authors observed in HFmrEF (24, 40). Conversely, the E/E′ ratio was in the normal range and remained unchanged after the CT program. In our opinion, this finding supports the hypothesis that an impaired LA reservoir strain is the first detectable sign of LV diastolic dysfunction. This result complies with the current literature: the LA reservoir strain alone or in combination with the E/E′ ratio has already been successfully tested as a single non-invasive index to predict the elevated LV filling pressures in patients with stable ICM and preserved LVEF (5, 41). Regarding the comparison between the two supervised CT regimens, our results suggest that there is a dose–effect relationship between the volume of exercise administered and the effect size produced on the LA function. It should be underlined that the same volume of exercise reached by the CTHF could have also been obtained by the patients from the CTLF group by increasing their session workload. There are clear demonstrations that, by equalizing the weekly training volume, different exercise training regimens produce similar effects on the skeletal muscle and cardiovascular parameters (42, 43). However, we were prevented from increasing the workload of the CTLF group by the fear of incurring muscle injuries and losing patient compliance. Interestingly, the effects of CT on LA function appeared to be uncoupled from those on exercise tolerance. It is noteworthy that the CTHF and CTLF groups obtained similar time increases during the exercise test at 12 weeks. This result seems to indicate that the volume of CT to be prescribed to patients with ICM could vary according to the different goals to be reached. Higher weekly volumes seem preferable when the object is that of inducing LA adaptations. In light of these observations, we believe that the results of this study go in the direction of a more personalized and individually tailored prescription of exercise training for patients with ICM. Further studies with longer training programs are needed for clarifying which is the best exercise intervention, in terms of modality, intensity, and duration, for eliciting LA adaptations.

### Limitations

The most important limitations of the present study are the small sample size and the short duration of the exercise protocol, which was limited to 12 weeks. New studies, including larger sample sizes and longer exercise training interventions, are needed to better understand the efficacy of CT in improving LA function in patients with ICM. The results of this study have been obtained by means of CT and they cannot be generalized to other exercise modalities. Because both groups of patients who underwent supervised training sessions performed the same protocol at each session, their weekly volume was not equated; therefore, we cannot rule out that in a scenario of equated weekly training volume the study would have produced different results. Finally, some caution in interpreting the data is needed because strain echocardiographic imaging has several technical limitations (44, 45), and the impairment in the myocardial strain parameters may not always be an expression of intrinsic myocardial dysfunction (46). Therefore, further confirmations of our results should be obtained with alternative diagnostic techniques, such as cardiac magnetic resonance imaging. Our data suggest that CT elicited LA adaptation in patients with ICM. However, this study does not contribute to the understanding of the mechanisms through which CT exerts its effects on LA, and this point needs to be clarified in further studies.

## Conclusion

This study showed that a 12-week CT program improved LA function in patients with ICM in a dose–effect manner. Further larger studies are needed to understand whether these exercise-induced benefits can be sustained over time and can translate into clinical benefits for patients with ICM.

## Data Availability

The original contributions presented in the study are included in the article/Supplementary Material, further inquiries can be directed to the corresponding author.
